# Self-Propelled Rotary Tools in Hard Turning: Analysis and Optimization via Finite Element Models

**DOI:** 10.3390/ma15248781

**Published:** 2022-12-08

**Authors:** Usama Umer, Syed Hammad Mian, Muneer Khan Mohammed, Mustufa Haider Abidi, Khaja Moiduddin, Hossam Kishawy

**Affiliations:** 1 Advanced Manufacturing Institute, King Saud University, Riyadh 11421, Saudi Arabia; 2 Machining Research Laboratory, University of Ontario Institute of Technology, Oshawa, ON L1G 0C5, Canada

**Keywords:** finite element (FE) models, self-propelled rotary tool (SPRT), hard turning

## Abstract

This study investigates self-propelled rotary tool (SPRT) performance in hard turning using 3D finite element (FE) models. The FE models developed in this study are based on coupled temperature-displacement analysis using an explicit time-integration scheme. The developed FE models can predict chip morphology, cutting forces, tool and workpiece stresses and temperatures. For model verification, hard turning experiments were conducted using an SPRT on AISI 4340 bars. Cutting forces and maximum tool–chip interface temperatures were recorded and compared with the model findings. The effects of different process parameters were analyzed and discussed using the developed FE models. The FE models were run with a central composite design (CCD-25) matrix with four input variables, i.e., the cutting speed, the feed rate, the depth of the cut and the inclination angle. Response surfaces based on the Gaussian process were generated for each performance variable in order to predict design points not available in the original design of the experiment matrix. An optimization study was carried out to minimize tool stress and temperature while setting limits for the material removal rate (MRR) and specific cutting energy for the process. Optimized processes were found with moderate cutting speeds and feed rates and high depths of cut and inclination angles.

## 1. Introduction

New materials have been proposed as a result of advances in material science. These contemporary materials have peculiar mechanical and thermal characteristics. They have a wide range of uses in high-performance industries, such as the biomedical field, electronics, aerospace, automobiles, etc., because of their exceptional qualities. However, while on one hand, these materials’ excellent attributes are useful, they really provide challenges and difficulties throughout the machining process. Excessive heat emission poses a significant obstacle in processing of these materials, since it affects tool wear and machining effectiveness [1,2,3]. In earlier studies, a variety of measures were employed to dissipate generated heat. For instance, coolant (or lubricants) have commonly been used to distribute and minimize the impact of produced heat, keeping the cutting-region temperature within a manageable threshold [4,5,6,7]. The lubricating properties of the oils and liquids utilized in the cutting zone serve to lessen the extent of the interaction between the chip and the tool and create a fine layer [8]. The positives of using coolant or lubricant during machining are obvious, but doing so has significant negative effects on both people and the environment [9]. In order to prevent harmful cutting fluids from being used during machining operations, experts have looked into the possibility of dry machining [10]. Hard turning and grinding are the two most common machining methods for materials that are challenging to work with [11]. Grinding has low throughput and limited possibilities in terms of versatility and machined shapes, according to Astakhov [12]. On the other hand, turning tough-to-cut materials rather than grinding them results in a high-quality machined surface at a reduced price [13]. In recent times, industry attention has increased regarding the use of turning without cutting fluids for hardened steel and other difficult-to-machine materials rather than grinding [14].

Hard turning refers to machining hard materials above 45 HRC: typically, hardened steels on a lathe or turning center. Hardened steels have exceptional properties in terms of abrasion resistance, durability, heat and corrosion resistance. They are a popular choice for automotive, aerospace and machine-tool parts. However, the tool-like properties of hardened steels generate high tool–chip interface temperatures that adversely affect tool performance and surface quality of the workpiece. Unlike conventional turning, hard turning poses a serious challenge in terms of process optimization that can improve workpiece surface characteristics and reduce tool wear. To address these issues, researchers have proposed various solutions, such as utilization of cutting fluids or machining with rotary tools. Despite considerable research that has been carried out on effective utilization of cutting fluids, its scope is limited due to environmental concerns. Severe tool wear has slowed down progress of hard turning [15,16]. Controlling tool wear and its effect on the uniformity of the workpiece surface has therefore been a significant technical challenge.

When conventional tools and materials are used, the cutting zone’s temperature rises, causing sudden tool wear that impairs the workpiece’s accuracy, surface integrity and tool geometry. For instance, carbides are often used at modest speeds for routine turning operations rather than hard turning to prolong tool life [17]. Furthermore, a single cutting-point tool with merely a single primary cutting edge has disadvantages when rigorous machining is performed. The problem is that the cutting tool will wear out quickly and become hot at the tip, which leads to early tool failure. The tool will rapidly increase in temperature throughout machining as a result of persistent physical contact with the workpiece, which speeds up tool wear and damages the machined surface thermally. Rotary cutting tools are the most popular option for machining hardened materials because of their capacity to maintain the performance of a usable cutting edge at high temperatures. Due to their enormous potential, rotary tools are an appealing option for manufacturing difficult-to-machine materials [18,19]. Rotary turning is a machining process that combines standard-turning tool operations with the rotary motion of a round turning insert [20]. A round cutting insert in a rotary tool spins about its main axis and can classify the tool as either an actively driven rotary tool (ADRT) or a self-propelled rotary tool (SPRT) [21,22]. Rotation to the cutting insert can be applied in two ways, i.e., either use of an electric motor driven by an external power supply (ADRT) or use of a high inclination angle between the rotating workpiece and the cutting insert without need for any external power (SPRT).

With an SPRT, tool rotational speed and direction depend on inclination angle, whereas ADRTs provide better control of tool rotational speed and direction, as the ADRT is independent of the tool-inclination angle. However, the need for external power sources for ADRTs leads to design complexity and results in expensive tooling. In contrast, SPRTs are easy to design and can provide low-cost solutions for rotating-tool applications. Nevertheless, their successful applications in machining require in-depth investigations with regards to the effect of a high inclination angle on different performance variables.

The literature has a plethora of articles by researchers that have conducted experimental evaluations to investigate the effectiveness of SPRTs in cutting challenging materials. For instance, experimental results for the machining of a SiCw/Al composite using carbide rotary cutting techniques, namely SPRTs, were published by Chen et al. [23]. Their research revealed that rotary tools had a tool life that was noticeably longer than that of existing tools. Similarly to this, the effects of SPRTs during the machining of aerospace material (Titanium IMI 318 alloy) were explored [24]. Comparing a cemented-carbide SPRT to round and rhomboid inserts used in single-point turning, the investigation showed that the cemented carbide SPRT extended tool life 60-fold. In a related study, Kishawy and Wilcox [25] evaluated SPRT effectiveness in machining of hardened steel. The carbide SPRT demonstrated exceptional wear resilience, negligible crater wear and evenly diffused flank wear. In order to assess SPRTs and surface quality, Kishawy et al. [26] also devised an experimental investigation. In that work, the full potential of SPRTs was seen when they were compared to a single-point tool with the same angle and tool profile. Additionally, SPRTs’ drawbacks (such as chips whirling across the tool’s rotating rake face) and benefits (such as longer tool life) were disclosed [27,28]. These experiments unquestionably demonstrated the usefulness and practicality of SPRTs for machining of challenging materials. Apparently, it is possible to conclude from the literature that SPRTs offer a number of benefits in processing of difficult-to-machine material.

The efficacy of SPRT machining can be impacted by a number of variables, including the cutting speed, the depth of the cut, the feed rate, tool wear, the tool geometry, etc. As a result, the literature can also be used to emphasize a number of studies that look at the various variables. As an example, Armarego and Katta [29] used SPRT machining to create a prediction model for forces and power during processing of S1214 steel. An SPRT mathematical model was developed for flank wear in a different investigation. A model for SPRT machining was also devised by Hao et al. [30], utilizing a combination of an artificial neural network (ANN) and a genetic algorithm (GA) to quantify cutting forces. The input parameters were feed rate, cutting velocity, cutting depth and tool inclination. When employing SPRTs during cutting, Hao et al. [31] proposed an autoregressive model to investigate incidence of vibration and chatter. The findings of this study showed that when the primary cutting power was dependent on a high frequency, excessive vibrations were generated. While using rotary and stationary tools in hard machining, Olgun and Budak [17] examined cutting forces, tool life, surface quality and dimensional accuracy. Gurgen et al. [22] utilized an SPRT to machine hardened steel and carried out multi-response optimization. Integrating the technique for order preference through similarity to ideal solution (TOPSIS) with non-dominated sorting genetic algorithm II (NSGA-II) allowed for modification of surface roughness and the material removal rate (MRR). Additionally, Kishawy [32] conducted a thorough review of SPRT machining and underlined the need for selecting the right machining and tool geometry settings to attain SPRTs’ exceptional functionality.

Despite the fact that a number of experimental research and mathematical models pertaining to application of SPRTs have been covered in earlier works, the literature unfortunately only contains a very small number of studies that have used the multi-objective optimization approach to the field of SPRT machining. Ahmed et al. [33], for instance, investigated how the inclination angle affected the integrity of the surface and tool wear. They used genetic programming and NSGA-II, artificial-intelligence-based techniques, to simulate and improve cutting of hardened steel using SPRTs. Tool wear, surface roughness and MRR were the analyzed outcomes, and the design factors chosen were the feed rate, cutting velocity and the inclination angle.

With the advancement of computational technologies, numerical methods are being extensively utilized in modeling and optimization of engineering problems. Among them, finite element methods have proven to be the most reliable and robust methods in solving complex real-world problems. For example, Rozylo [34] performed topology optimization for an I-section profile in an effort to reduce its volume while putting a limit on the maximum stress generated and displacement using an ABAQUS^®^ optimization module.

Unlike other studies based on empirical models, the aim of the current study was to examine SPRT performance in hard turning, using FE models in order to save costly experimental runs. Up until now, no study has been found that was based on multi-objective and multi-constraint optimization utilizing 3D FE models with realistic cutting geometries in machining of hardened steel with an SPRT. These models are also able to predict changes in chip morphologies with varying cutting parameters. In this study, a 3D FE model based on coupled temperature-displacement analysis was developed using an explicit time-integration scheme. Chip-formation analysis for had turning using SPRT was carried out, and the model’s outcomes were compared with experimental results. The verified FE models were run for a design of experiment (DOE) matrix to find SPRT performance variables. Use of model findings and response surface multi-objective optimization for SPRT machining was carried out to minimize tool stress and temperature while setting constraints for the material removal rate and specific cutting energy.

## 2. Materials and Methods

### 2.1. Experimental Setup

**Figure 1:**
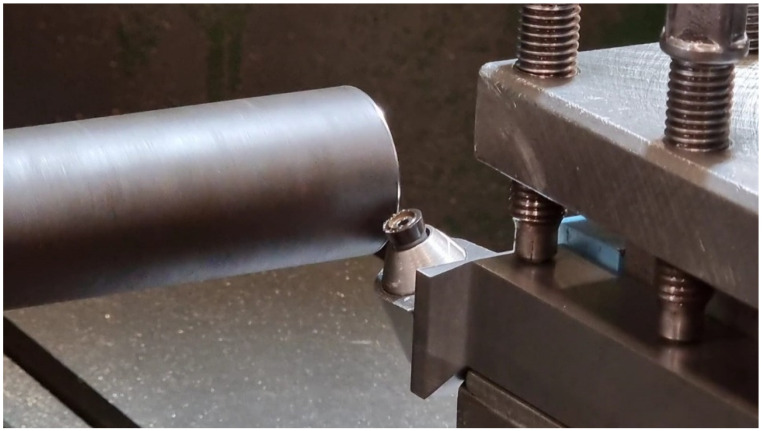
Turning experiment setup with SPRT.

Hard turning experiments were conducted on AISI 4340 with 55 HRC, using round coated-carbide inserts and a rotary tool holder, as shown in Figure 1. The cutting parameters for the turning process and tool geometric parameters are shown in Table 1. The rotary tool holders were developed in-house with three inclination angles, as shown in Table 2. Sandvik Coroturn^®^ RCMT (Sandvik AB, Stockholm, Sweden) type cutting inserts with a KCP25 material grade were employed. KCP25 is a tough cobalt-enriched grade with multilayer TiCN-Al_2_O_3_ coatings with superior interlayer adhesion. For optimization using the developed 3D FE model, a central composite design (CCD-25) was selected. A total of 8 design points from the CCD were chosen to verify the FE model using cutting-force data and maximum tool–chip interface temperature. A piezoelectric dynamometer (Kistler 9257B, Winterthur, Switzerland) was employed to measure forces in cutting and radial direction. The maximum tool-interface temperature was captured using a thermal imaging camera (Optris 640, Berlin, Germany) and mounted on the tool post.

### 2.2. The Finite Element Model for SPRTs

**Figure 2:**
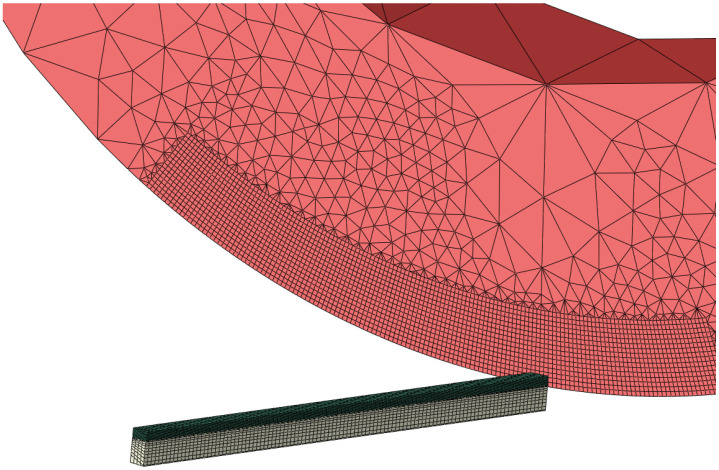
Undeformed mesh for cutting tool and workpiece.

The finite element SPRT model was developed using ABAQUS/CAE, as shown in Figure 2. In order to reduce computational time, only a part of the cutting insert was made deformable, while other elements were constrained as rigid body elements. A small portion of the workpiece was considered for modeling and allowed to move in the cutting direction only. The cutting insert was allowed to rotate along the central axis in the cutting direction only while all other degrees of freedom were constrained. Except for the rigid part of the cutting insert, hexahedral elements with reduced integration and temperature degree of freedom were selected for the cutting tool and the workpiece. For the rigid part of the cutting tool, tetrahedral and coarser elements were selected to reduce model size. To simulate high temperature and stress gradients, a very fine mesh was designed for the deformable part of the cutting tool, as shown in Figure 2.

AISI 4340 is considered an elasto-plastic material with rate-dependent plasticity behavior and thermal softening effects. The Johnson–Cook model, which is mostly employed in machining and high rate-forming, was adopted here for the 3D FE SPRT model, and flow stress (σ) is given with:

(1) $$\sigma =\left(A+B\varepsilon^{n}\right)\left(1+C\ln\left(\frac{\dot{\varepsilon }}{{\dot{\varepsilon }}_{0}}\right)\right)\left(1-{\left(\frac{T-T_{r}}{T_{m}-T_{r}}\right)}^{m}\right).$$

Flow stress was calculated based on current strain (ε), the strain rate ($\dot{\varepsilon }$) and the temperature (T). In Equation (1), A refers to the initial yield strength of the material at the reference strain rate, *B* is the strain-hardening coefficient, *n* is the strain-hardening exponent, *C* is the strain-rate coefficient, *m* is the thermal-softening exponent, ${\dot{\varepsilon }}_{0}$ is the reference strain rate, $T_{r}$ is the reference for room temperature and $T_{m}$ is melting temperature. The constants (*A*, *B*, *n*, *C* and *m*) can be determined experimentally with standard high-strain-rate testing, e.g., the split Hopkinson bar test.

Chip separation from the workpiece was modeled using an element-deletion technique through addition of a sacrificial layer between the workpiece and the chip. The elements were deleted according to Johnson–Cook damage parameter ‘*D*’, which is defined as:

(2) $$D=\sum \frac{\Delta \varepsilon }{\varepsilon_{f}},$$

where Δε is the incremental plastic strain of each element in the sacrificial layer and $\varepsilon_{f}$ is the plastic strain at fracture. This is defined using Johnson–Cook damage constants (*D*_1_ to *D*_5_) and depends on strain, the strain rate and temperature. As per Johnson–Cook damage law, $\varepsilon_{f}$ is given with:

(3) $$\varepsilon_{f}=(D_{1}+D_{2}\exp\left(D_{3}\sigma^{*}\right)\left(1+D_{4}\ln\left(\frac{\dot{\varepsilon }}{{\dot{\varepsilon }}_{0}}\right)\right)\left(1-D_{5}{\left(\frac{T-T_{r}}{T_{m}-T_{r}}\right)}^{m}\right),$$

where $\sigma^{*}$ is the ratio of pressure to von Mises stress. The damage parameter ‘*D*’ is evaluated for each element, and fracture is initiated when *D* = 1. The Johnson–Cook flow and damage parameters are shown in Table 2 [35].

After verification from experiments, these FE models were used for the CCD matrix. The models were evaluated based on four output variables, i.e., tool stress (S), the tool temperature (T), specific cutting energy (U) and the material removal rate (MRR). Based on the outcomes of the CCD matrix, response surfaces based on the Gaussian process were developed for each output variable. These response surfaces were used as input for the optimization solver for design points not available in the CCD matrix.

### 2.3. Optimization Problem

**Figure 3:**
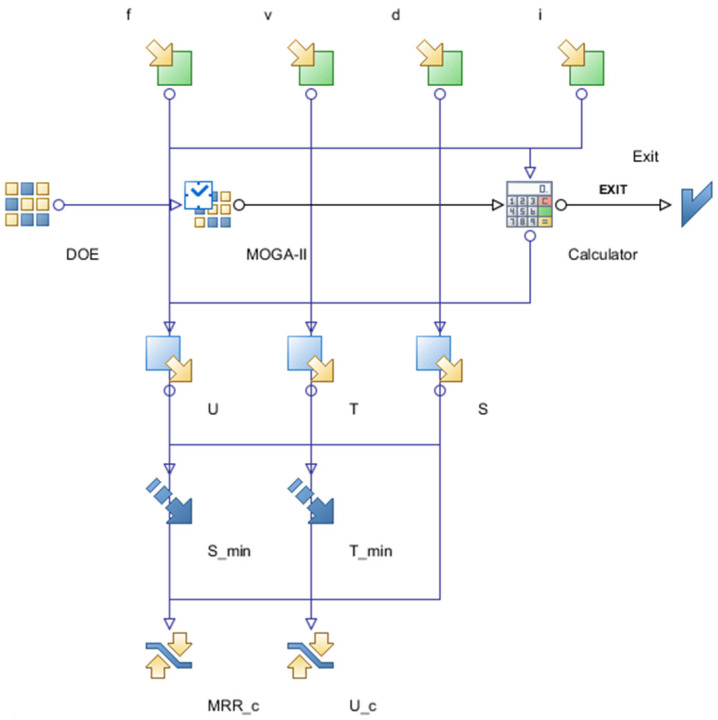
Optimization workflow showing all input and output variables.

The optimization problem was set to minimize the maximum tool temperature and stress while keeping a limit on specific cutting energy and the material removal rate. The optimization problem is shown in Table 3. Regression modeling and optimization work were carried out using optimization program MODE FRONTIER^®^, developed by Esteco SpA (Trieste, Italy). For optimization search, a multi-objective genetic algorithm, MOGA-II, was adopted; it is based on smart multi-search elitism for robustness and directional crossover. The optimization workflow, containing all input variables, output variables, objectives and constraints, is shown in Figure 3.

## 3. Results and Discussion

Table 4 shows the results of cutting forces and maximum tool–chip interface temperatures obtained from experiments and FE models for selected design points from the CCD matrix. Good agreement can be noticed between experiments and model results. Dependencies of cutting force and temperature on each input variable, as predicted by the FE models, were found to be similar to the experimental results, as shown in Table 4. The average errors for cutting force and temperature were found to be around 10% and 12%, respectively. In all cases, the FE models underestimated cutting force and temperature. This could have been due to various assumptions taken during the development of these FE models, such as absence of tool wear, continuous chip formation and constant coefficient of friction at the tool–chip interface.

**Figure 4:**
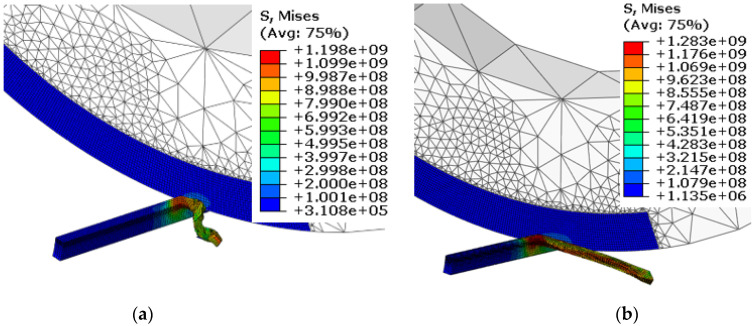
Chip formation and von-Mises-stress contours for SPRT and workpiece at different feed rates: (**a**) f = 0.1 mm/rev, (**b**) f = 0.2 mm/rev (V = 80 m/min, d = 0.3 mm and i = 17°).

The chip-formation and von-Mises-stress results for the workpiece and the cutting tool at different feed rates are shown in Figure 4. The chip morphology and the von-Mises-stress contours on the workpiece appeared to be very different between both feed rates. Evidently, low feed rates resulted in chip curling and consequently showed alternate bands of high and low von Mises stress at the chip surface. In contrast, higher feed rates resulted in straight chips with uniform stress distribution. Chip curling/chip segmentation with low feed rates have been observed by many researchers. Low feed rates result in low chip–tool contact length, resulting in a higher chip-flow angle that leads to chip curling. This phenomenon can be well understood through comparison of tool–chip contact length for both feed rates in Figure 4.

**Figure 5:**
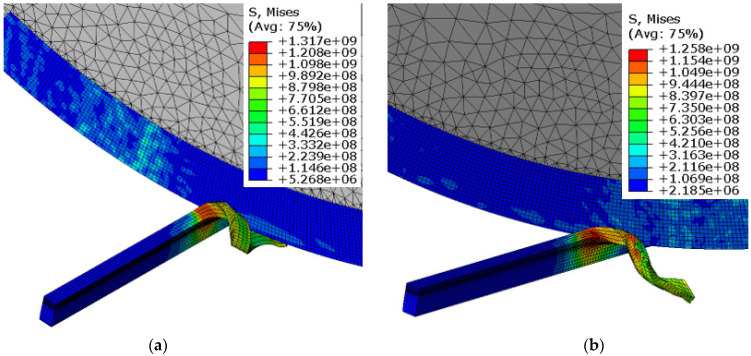
Chip formation and von-Mises-stress contours for SPRT and workpiece at different inclination angles: (**a**) i = 9°, (**b**) i = 25° (V = 100 m/min, f = 0.1 mm/rev, d = 0.3 mm).

Figure 5 shows the chip morphology and the stress contours for the cutting tool and the workpiece at different inclination angles. Again, chip morphology and stress contours were found to be very different with changes in the inclination angle, as shown in Figure 5. Chip curling reduced as the inclination angle was increased from 9° to 25°. This is attributed to variation in chip contact length and chip flow angle, which also results in reduction in von Mises stress. As shown in a previous study [36], cutting force fluctuates with the inclination angle due to changes in direction of the resultant force acting on the chip. This can also be visualized from Figure 5, as the height of the tool–chip contact area was reduced when the inclination angle was increased from 9° to 25°. Similar observations were made in a recent study [33] when AISI 4140 was machined with an SPRT. The researchers noticed cutting-force fluctuations with changes in both the cutting speed and the inclination angle.

**Figure 6:**
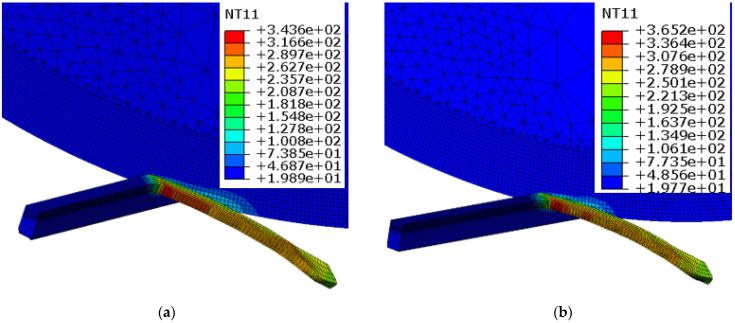
Chip formation and temperature contours for SPRT and workpiece at different cutting speeds: (**a**) V = 80 m/min, (**b**) V = 100 m/min (f = 0.1 mm/rev, d = 0.3 mm, i = 17°).

The chip formation and the temperature contours of the cutting tool and the workpiece at different cutting speeds are shown in Figure 6. A rise in temperature is obvious with higher cutting speeds: a well-known phenomenon in metal cutting. Higher cutting speeds resulted in an escalation of the rate of plastic deformation, which resulted in higher heat dissipation, as depicted in Figure 6. In addition, the high-temperature area of the SPRT noticeably reduced with the cutting speed. This occurs simply because less time is available for heat transfer between the cutting tool and the workpiece at higher cutting speeds. A similar observation can also be made regarding outer chip-surface temperature. Magnitude appeared to be high at the primary shear zone, and area reduced with a rise in the cutting speed. In contrast to the fixed-tool case, heat was concentrated at the cutting zone, and temperatures were much higher, as reported by Ahmed et al. [37] in a comparative study.

**Figure 7:**
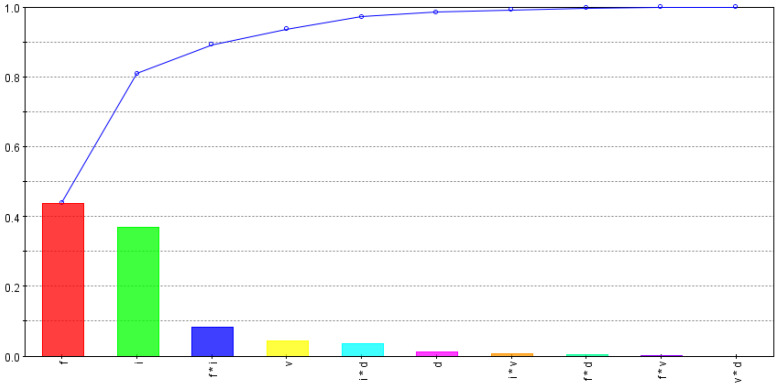
Tool-stress dependencies on input variables and their interactions.

**Figure 8:**
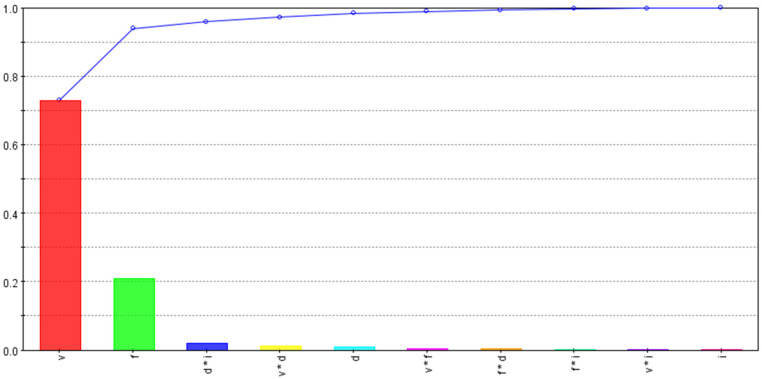
Tool-temperature dependencies on input variables and their interactions.

**Figure 9:**
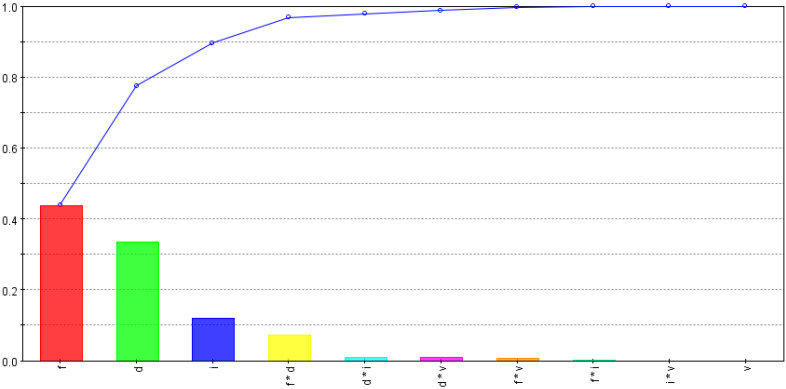
Specific-cutting-energy dependencies on input variables and their interactions.

The developed 3D FE models were run for the CCD-25 matrix, and the maximum tool temperature and stress were noted for each run, along with the cutting force, to evaluate specific cutting energy for the process. Table 5 shows all of the input and output variables used for the optimization study. The percentage contributions of input variables and their interactions for each performance variable were evaluated using smoothing spline analysis of a variance algorithm and are shown in Figure 7, Figure 8 and Figure 9.

Figure 7 shows effects of input variables and their interactions on maximum tool stress. The feed rate and the inclination angle were found to be significant contributors to changes in tool-stress values, followed by the cutting speed and the depth of the cut. As discussed earlier, both the feed rate and the inclination angle affect tool–chip contact area and shape; tool stresses are likely to fluctuate with changes in either variable and also with their interaction. As the cutting speed did not affect shear area and cutting-force changes were minimal, their effect on tool stress was low, as shown in Figure 7. Tool-stress dependency on the depth of the cut was found to be negligible, as change in uncut shear area was almost proportional to change in cutting force.

The percentage contributions of input variables and their interactions to maximum tool temperatures are shown in Figure 8. As expected, the tool temperature was found to be largely dependent on the cutting speed, followed by the feed rate. The effects of the depth of the cut and the inclination angle were very low, as shown in Figure 8. This may be due to the balancing effect, i.e., a rise in temperature via the depth of the cut being partially nullified by a decrease in the tool temperature as the tool’s rotational speed increases with the inclination angle. This was also observed by Ahmed et al. [37] when they modeled turning of AISI 1045 using an SPRT. It should be noted that the reduction in temperature with the rise in the tool rotational speed has a certain limit. With higher rotational speeds, the contact time between the SPRT and the chip is reduced, resulting in an increase in tool temperature.

Figure 9 shows the effects of input variables and their interactions on the specific cutting energy of the process. The feed rate and the depth of the cut were found to be the most influential, followed by the inclination angle and the combined effect of the feed and the depth of the cut. As specific cutting energy depends on the uncut chip area and cutting force, the higher effects of the feed rate and the depth of the cut were obvious. The dependency of specific cutting energy on the inclination angle is simply due to changes in chip–tool contact area or cutting-force fluctuations.

**Figure 10:**
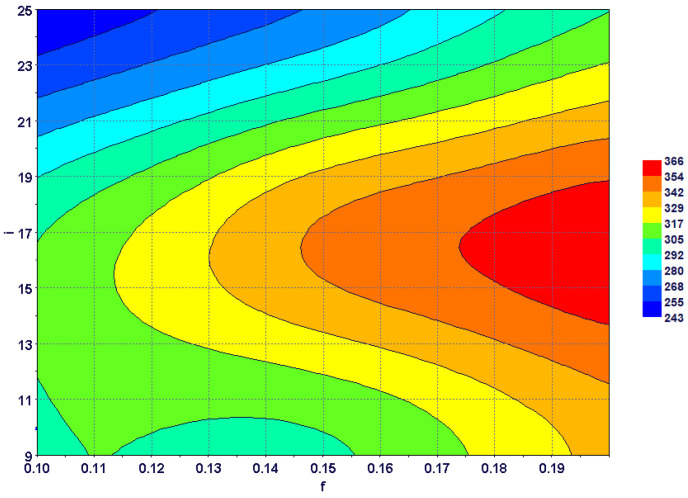
Combined effect of feed rate and inclination angle on maximum tool stress.

The combined effect of the feed rate and the inclination angle on maximum tool stress is shown in Figure 10. It is clear that increasing the feed rate gave rise to higher tool stress at all inclination angles. However, the rate of variation changed with the inclination angle value, and rapid changes could be seen at higher inclination angles. Tool stress first increased and then decreased with inclination angles at all feed rates. Maximum tool stress could be seen at high feed rates and moderate inclination angles. This can be correlated with cutting-force variations with inclination angles, presented in an earlier study [36].

**Figure 11:**
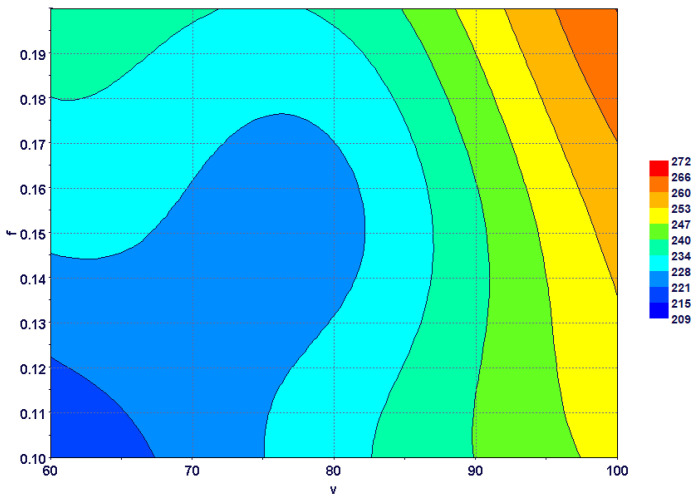
Combined effect of cutting speed and feed rate on maximum tool temperature.

Figure 11 shows the combined effect of the cutting speed and the feed rate on the maximum tool temperature. The tool temperature increased with both the cutting speed and the feed rate, and the maximum could be found at high cutting speeds and feed rates. The effect of the cutting speed was more pronounced compared to that of the feed rate. This is because at higher cutting speeds, less time is available for heat transfer to the chip, the workpiece and the tool body. In contrast, increasing the feed rate also increased uncut chip thickness, i.e., more heat could be carried out by the chip at the same cutting speed.

**Figure 12:**
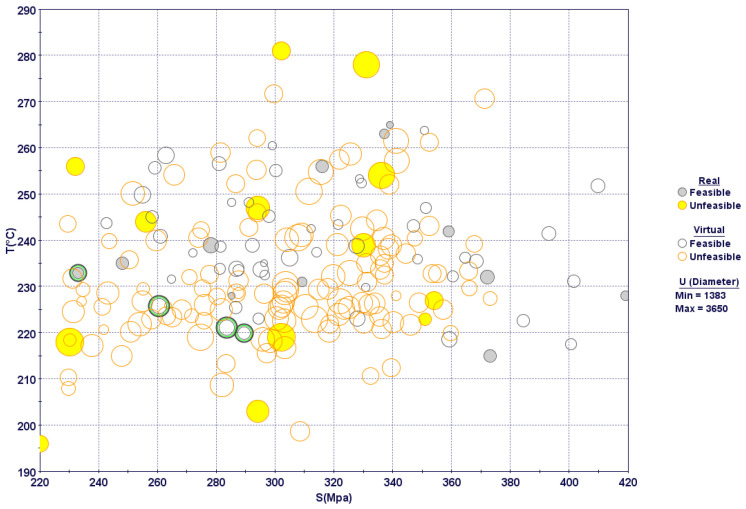
Three-dimensional bubble chart showing design points after optimization search (Green circles represent optimum design points).

A 3D bubble chart with all of the design points obtained after optimization runs is shown in Figure 12. Real design points are experimental runs shown in the CCD-25 matrix, whereas virtual design points are predicted by the response surfaces developed for each performance variable. Unfeasible design points, i.e., experimental runs that violated the constraint set in the optimization problem, are shown in yellow. As per the design objectives, the design points on the lower left portion should be considered experimental runs with optimal parameters. From a look at the feasible design points, four points can be marked as optimal (marked with green inner circles) on the Pareto mode front, as shown in Figure 12. For other design points in the lower left corner, most constraint violations occurred due to a low MRR. Similarly, most of the design points on the right hand side became unfeasible due to high specific cutting energies. The optimal design points selected had mostly moderate cutting speeds, moderate feed rates, higher depths of cut and higher inclination angles. Details of the optimal solutions are shown in Table 6.

**Figure 13:**
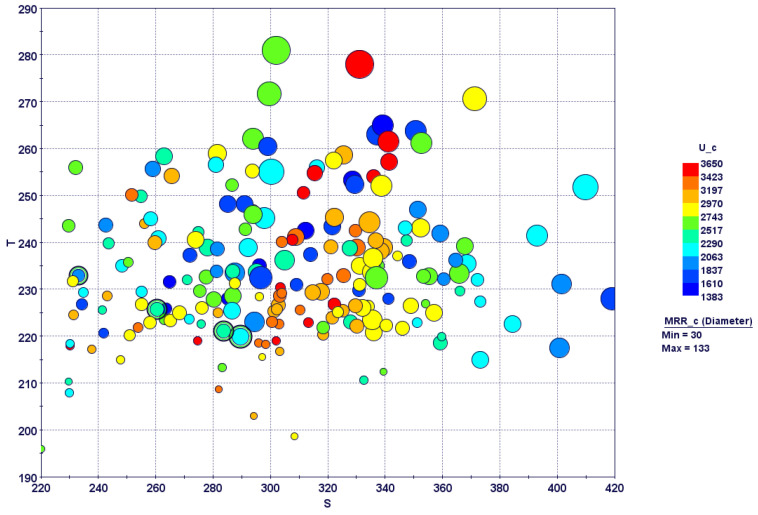
Four-dimensional bubble chart with optimum design points.

Figure 13 shows the same design points plotted against the two objectives, with information regarding the constraints, i.e., specific cutting energy and the MRR. It is obvious that the lower left portion is mostly occupied by low material-removal rates and moderate specific cutting energies. These experimental runs were mostly associated with low cutting speeds and feed rates. The design points on the extreme right, however, fell in the feasible range; they were associated with very high stresses due to larger feed rates. Similarly, the design points at the top were associated with higher specific cutting energies, as cutting forces were higher at low and moderate inclination angles.

**Figure 14:**
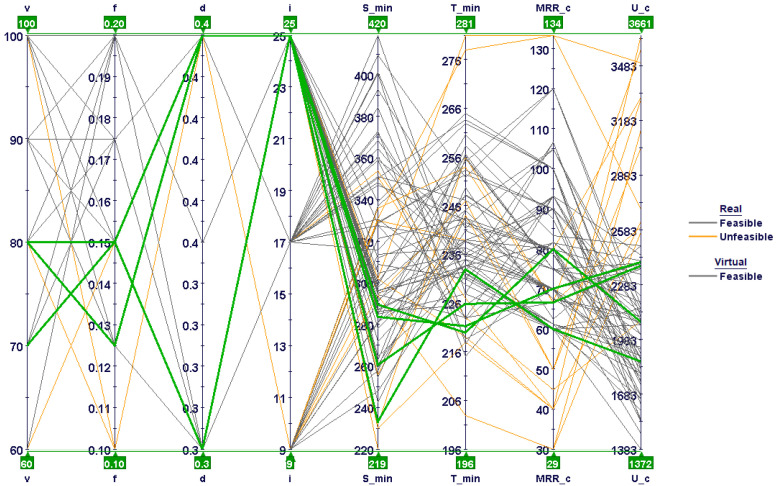
Parallel coordinate charts showing all design variables.

A parallel coordinate chart reveals all design variables at a time, shown in Figure 14. The optimum design points are shown with green lines. Evidently, optimum design points were mostly associated with low cutting speeds and feed rates and with higher depths of cut and inclination angles. Lower feed-rate values mostly led to unfeasible design points due to high specific cutting energies, as shown in Figure 14. In addition, most of the design points with low to moderate inclination angles were characterized with high tool stresses and temperatures, including both feasible and unfeasible points. These design points are located in the middle and on the right side of the bubble charts illustrated in Figure 12 and Figure 13, respectively.

## 4. Conclusions

In this study, FE models were developed for optimization in hard turning processes that use SPRT machining. As turning with SPRTs is carried out with an oblique cutting geometry, 3D models with a realistic tool geometry are indispensable. The developed models were based on a complete cutting-insert geometry that can be used to optimize temperature using ABAQUS/standard heat-transfer analysis. The developed models showed the capability to simulate important performance indicators, such as cutting forces and maximum tool–chip interface temperature, with a reasonable degree of accuracy. In addition, the developed models could be unitized to predict residual stress profiles along and beneath the machine surface through addition of the tool-unloading and cooling-cycle steps. In addition, these models can be used to predict machining-process quality through observation of chip morphology, as discussed in the Results section. The following conclusions can be drawn from this study:

1. The 3D FE models developed for the SPRT show good agreement with the experimental results, and the errors were 10% and 12% for cutting force and temperature, respectively.
2. These models are able to predict changes in chip morphology with varying feed rates.
3. Low feed rates result in higher chip flow angles, which leads to chip curling.
4. Cutting force and von Mises stress fluctuate with the tool inclination angle. Low von Mises stress is found at higher inclination angles.
5. Higher cutting speeds result in escalation of maximum tool–chip interface temperatures.
6. Tool stress is found to be highly dependent on the feed rate and the inclination angle, whereas the effects of the cutting speed and the depth of the cut are found to be negligible.
7. Optimized SPRT performance is found with moderate cutting speeds and moderate feed rates, with higher depths of cut and higher inclination angles.

## Figures and Tables

**Table 1:** Variables of cutting and tool geometry for experiments and finite element (FE) models.

Cutting Parameter	
Speed (V)	60, 80 and 100 m/min
Feed rate (f)	0.1, 0.15 and 0.2 mm/rev
Depth of cut (d)	0.3 and 0.4 mm
Cutting-Tool Geometry	
Rake angle	−15°
Clearance angle	5°
Inclination angle	9°,17° and 25°
Diameter	27 mm
Edge radius	0.05 mm

**Table 2:** Johnson–Cook’s flow and damage parameters.

J–C Flow Model	*A* (MPa)	*B* (MPa)	*C*	*n*	*m*
	792	510	0.26	0.014	1.03
J–C Damage Model	*D*_1_	*D*_2_	*D*_3_	*D*_4_	*D*_5_
	0.05	3.44	−2.12	0.002	0.61

**Table 3:** Optimization problem.

Objectives	1. Minimize S (MPa) 2. Minimize T (°C)
Constraints	1. U ≤ 2500 (J/mm^3^) 2. MRR ≥ 60 (mm^3^/s)

**Table 4:** Cutting force and temperature from experiments and simulations.

S. No.	V (m/min)	f (mm/rev)	d (mm)	i (°)	F_c_exp_ (N)	F_c_sim_ (N)	T_max_exp_ (°C)	T_max_sim_ (°C)
1	60	0.1	0.3	9	105	94	363	313
2	60	0.1	0.3	25	85	77	336	298
3	60	0.15	0.3	17	109	95	381	332
4	80	0.15	0.3	17	108	97	393	343
5	80	0.15	0.4	17	141	124	364	325
6	80	0.2	0.4	17	159	146	393	337
7	100	0.2	0.4	9	308	280	488	425
8	100	0.2	0.4	25	234	216	476	427

**Table 5:** CCD-25 matrix with input and output variables from SPRT models.

S. No.	V (m/min)	f (mm/rev)	d (mm)	i (°)	S (MPa)	T (°C)	U (J/mm^3^)	MRR (N/mm^3^)
1	60	0.1	0.3	9	294	203	3133	30
2	60	0.1	0.3	25	220	196	2575	30
3	60	0.2	0.3	9	309	231	1800	60
4	60	0.2	0.3	25	285	228	1383	60
5	60	0.1	0.4	9	302	219	3650	40
6	60	0.1	0.4	25	230	218	3650	40
7	60	0.2	0.4	9	330	239	3310	80
8	60	0.2	0.4	25	278	239	2342	80
9	60	0.15	0.3	17	351	223	2089	45
10	80	0.15	0.3	17	372	232	2156	60
11	80	0.15	0.4	17	373	215	2067	80
12	80	0.1	0.3	17	354	227	2633	40
13	80	0.2	0.3	17	359	242	1850	80
14	80	0.15	0.3	9	248	235	2067	60
15	80	0.15	0.3	25	233	233	1866	60
16	80	0.2	0.4	17	419	228	1825	107
17	100	0.1	0.3	9	294	247	3300	50
18	100	0.1	0.3	25	256	244	3033	50
19	100	0.2	0.3	9	337	263	1750	100
20	100	0.2	0.3	25	339	265	1533	100
21	100	0.1	0.4	9	336	254	3575	67
22	100	0.1	0.4	25	232	256	2600	67
23	100	0.2	0.4	9	331	278	3500	133
24	100	0.2	0.4	25	302	281	2700	133
25	100	0.15	0.3	17	316	256	2067	75

**Table 6:** Details of optimal solutions.

S. No.	V (m/min)	f (mm/rev)	d (mm)	i (°)	S (MPa)	T (°C)	U (J/mm^3^)	MRR (N/mm^3^)
1	80	0.15	0.3	25	233	233	1866	60
2	80	0.12	0.4	25	260	226	2389	67
3	70	0.15	0.4	25	284	221	2408	70
4	80	0.15	0.4	25	289	220	2082	80
